# Thermal stress depletes energy reserves in *Drosophila*

**DOI:** 10.1038/srep33667

**Published:** 2016-09-19

**Authors:** Peter Klepsatel, Martina Gáliková, Yanjun Xu, Ronald P. Kühnlein

**Affiliations:** 1 Max-Planck-Institut für biophysikalische Chemie, Research Group Molecular Physiology, Am Faβberg 11, D-37077 Göttingen, Germany

## Abstract

Understanding how environmental temperature affects metabolic and physiological functions is of crucial importance to assess the impacts of climate change on organisms. Here, we used different laboratory strains and a wild-caught population of the fruit fly *Drosophila melanogaster* to examine the effect of temperature on the body energy reserves of an ectothermic organism. We found that permanent ambient temperature elevation or transient thermal stress causes significant depletion of body fat stores. Surprisingly, transient thermal stress induces a lasting “memory effect” on body fat storage, which also reduces survivorship of the flies upon food deprivation later after stress exposure. Functional analyses revealed that an intact heat-shock response is essential to protect flies from temperature-dependent body fat decline. Moreover, we found that the temperature-dependent body fat reduction is caused at least in part by apoptosis of fat body cells, which might irreversibly compromise the fat storage capacity of the flies. Altogether, our results provide evidence that thermal stress has a significant negative impact on organismal energy reserves, which in turn might affect individual fitness.

Temperature is ecologically the most important physical factor that has significant effects on the physiology of organisms, particularly ectotherms^1^, and which is a key determinant of species geographical distribution and abundance^2^. Thus anthropogenic global warming, which proceeds at an unprecedented rate, presents a considerable challenge for many species^3^. Organisms have to adapt not only to the rising mean temperatures but also to more pronounced short-term temperature fluctuations^4^. Accuracy of the estimates of biotic changes in response to climate change is thus dependent on our understanding of how thermal stress affects animal physiology.

Environmental stress is defined as any event that threatens or disturbs physiological homeostasis and reduces the performance and fitness of organisms (reviewed in ref. 5). Depending on the duration, stress is classified as acute (short duration, minutes to hours) or chronic (long duration, days to weeks)^6^. In natural environments, exposures to different stressors are inevitable^7^, with acute stressors being more frequent than chronic stressors^6^. The overall effect of stress on an organism depends on its intensity and duration, meaning that long-term exposure to a relatively mild stress might be similarly detrimental as short-term exposure to a strong stressor^8^. On a cellular level, stress triggers a cellular stress response, which is a universal defence mechanism that is activated in response to macromolecular damage^9^. The role of the stress response is to mitigate stress-induced damages and to allow a restoration of cellular homeostasis. An important and evolutionarily conserved part of the cellular stress response is the activation of heat-shock proteins (Hsps)^9^,^10^. Heat-shock proteins bind to unfolded proteins and either assist in their refolding, prevent their nonspecific aggregation, or target them for degradation (reviewed in ref. 11). If the environmental stress overburdens the cellular defence mechanisms, macromolecules and cellular structures are irreversibly damaged and cells undergo apoptosis (reviewed in ref. 12).

The concept of energy-limited tolerance to stress^13^ postulates that stressful conditions affect energy allocation by modulating energy demands for different processes. Animals obtain energy in the form of chemical energy, which is spent on three different types of physiological processes: biosynthesis (e.g. growth, reproduction, energy deposition), maintenance, and external work^14^. In order to increase fitness, the acquired energy must be optimally allocated among these three different processes^15^. Under optimal environmental conditions, energy supply is sufficient to cover all somatic maintenance costs, and additionally it allows growth, reproduction, and/or accumulation of energy reserves. Under stressful conditions, however, maintenance takes priority to secure survival, while the rerouted energy flux causes growth arrest, paused reproduction, and mobilization of energy stores^13^.

The energetic status of an animal can be inferred from the amount of its energy reserves. Positive energy balance increases storage, whereas negative energy balance depletes energy deposits. The quantity of body energy reserves is an important and in many cases an essential component of organismal fitness. Natural populations are often exposed to food limitation due either to scarcity of nutrients or to intraspecific competition and accordingly, starvation is a major cause of mortality in many species (e.g. refs 16,17). Animals store energy in the form of complex carbohydrates (glycogen) and neutral lipids (fat). Fat reserves have several advantages over glycogen storage; the energy content of fats is more than double that of glycogen, and lipids are not hydrated. Thus, fat reserves contain eight to nine times more energy per unit mass than glycogen reserves^18^. Consequently, lipid reserves play the predominant role in resistance to starvation^19^. Carbohydrates (glucose, trehalose and glycogen), on the other hand, are the primary source of energy for flight in dipteran insects^20^. In *Drosophila* adults, the amount (mass) of glycogen and lipids is in the same order of magnitude^21^. Nevertheless, little is known about the effect of stress on the energy reserves.

In our study, we examined how chronically elevated temperature and acute thermal stress modulate energy reserves in *D. melanogaster*. We show that increased temperature affects both lipid and carbohydrate reserves, but body fat stores are more temperature-sensitive. Interestingly, short-term exposure to extreme temperatures has a similar fat-decreasing effect to long-term exposure to higher temperature. In contrast to starvation, the effect of thermal stress on lipid storage is long-lasting, extending far beyond the exposure period. We provide evidence that at least part of this long-lasting decline of body fat storage is due to the apoptosis of fat body cells, which is likely caused by irreversible temperature-induced damage. Our results suggest that in natural environments with fluctuating food availability, a loss of energy reserves due to thermal stress might have important fitness consequences.

## Results

### Ambient temperature affects *Drosophila* body fat content

**Figure 1:**
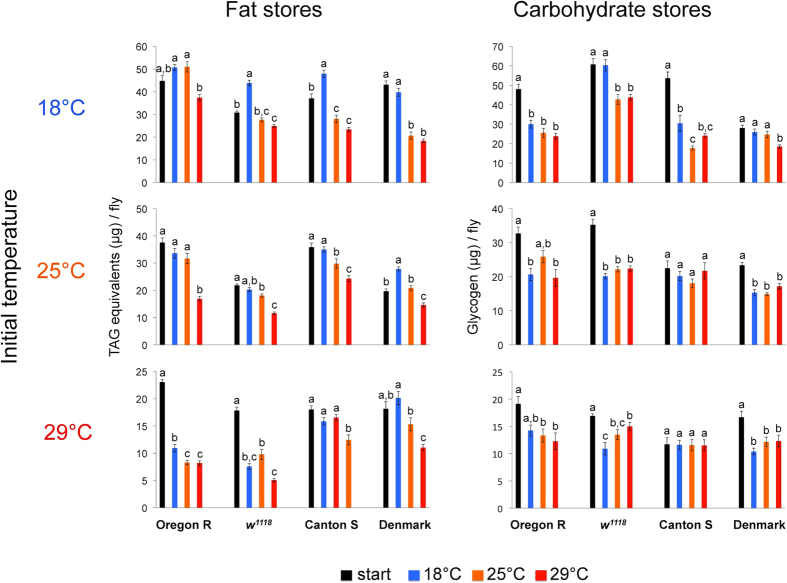
Temperature has significant effect on the fat reserves. Start values were measured at the beginning of the experiment. Data (for given population and initial temperature) were analysed by one-way ANOVA with Tukey’s HSD test: *P* < 0.05. Values marked with different letters are significantly different. Error bars represent standard errors of the mean. For global statistical analyses see Supplementary Tables S1 and S3.

In order to investigate how temperature affects *Drosophila* body energy reserves, we examined the temperature-induced changes in total fly lipid and glycogen storage of two different commonly used wild-type lab strains (Oregon R, Canton S) and a lab strain carrying a mutant allele for the eye colour gene *white (w*^*1118*^). To address possible confounding (detrimental) effects of inbreeding on stress resistance (reviewed in ref. 7), we also tested one wild-caught outbred population (Denmark^22^). We used a full factorial experimental design with three ‘initial’ temperatures (exposure during development + four days of adulthood) and three ‘adulthood’ temperatures (exposure time of eight days) (Supplementary Fig. S1). To exclude the possibility that the observed effects are caused by a specific diet by temperature interaction, the whole experiment was performed on two diets with different protein content. We found that on both diets, population (stock), initial and adulthood temperatures, and their interactions all have significant effects on glycogen and fat content (Supplementary Tables S1–4). Follow-up analyses of individual *Drosophila* strains from different initial temperatures confirmed that the fat reserves are strongly affected by adulthood temperature; male flies have significantly lower fat content at higher temperatures (Fig. 1, Supplementary Fig. S2). In contrast, the effect of adulthood temperature on glycogen content is rather weak (Fig. 1, Supplementary Fig. S2). Since the fat stores are the calorically more important body energy reserve and showed strong temperature-responsiveness, we focused our further investigation on this energy substrate.

Although our analyses of effects of initial temperatures (and populations) on the relative changes in the body fat content at three adulthood temperatures revealed a significant effect of initial temperature (Supplementary Tables S5 and S6), we did not find clear evidence for a positive effect of developmental acclimation on the relative changes in the body fat content (Supplementary Figs S3 and S4). In other words, we did not observe an unambiguous relationship (one that would hold for all analysed populations) between the extent of body fat depletion at any given adulthood temperature and the initial temperature. This suggests that there is rather a genotype-dependent modulation of the temperature-responsiveness of body fat content.

Next, we addressed the effect of developmental temperature on body fat content immediately after eclosion. Interestingly, the analysis of freshly eclosed flies showed that body fat content is lowest at developmental temperature 25 °C compared to 18 °C and 29 °C (Supplementary Fig. S5, Supplementary Table S7). Together with the previous results, this indicates that the major factor causing the inverse relationship between temperature and body fat reserves observed in older *Drosophila* adults is the ambient temperature during adulthood.

**Figure 2:**
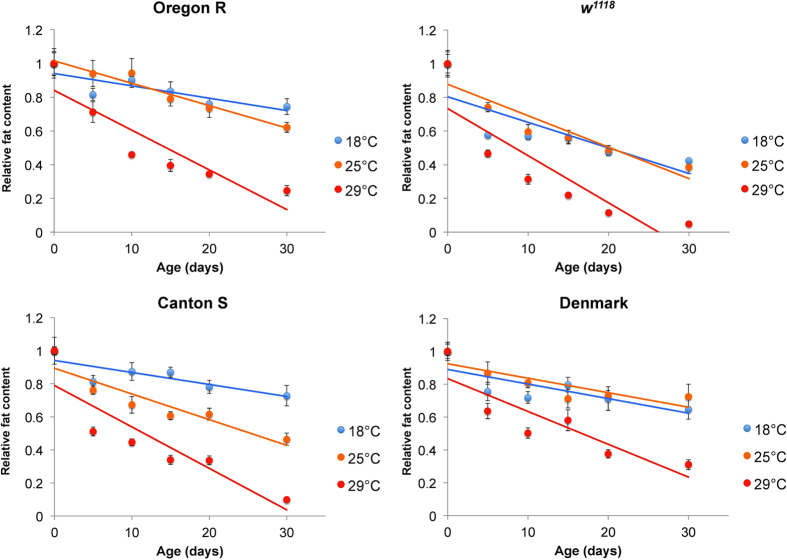
Age-related changes in the fat content at three temperatures. Data are standardized to the values at eclosion (0–24 h). Lines represent linear regressions. Error bars represent standard errors of the mean. For statistical analyses see Supplementary Table S8.

Furthermore, since it has been shown that aging affects *Drosophila* starvation resistance^23^, which might be caused by changes in the body fat content, we also examined the age-related changes in body fat reserves. To investigate the effect of varying ambient temperature on the body fat content of aging flies as well, we measured body fat content during the first 30 days of adulthood at three different temperatures (18 °C, 25 °C, 29 °C). We found a gradual age-dependent decrease in the fat content at all temperatures analysed (Fig. 2), which is faster at higher temperature (Supplementary Table S8). In addition, comparing the subcuticular abdominal fat body between young (4 days old) and aged (31 days old) flies revealed an apparent decrease in the fat body volume with age (Supplementary Fig. S6).

Finally, to confirm that the observed changes in the body fat content in response to temperature are based on triacylglycerols (TAGs), the major form of storage lipids, we performed thin layer chromatography (TLC). The TLC analysis confirmed that the temperature-induced changes in the lipid content are indeed caused by quantitative changes in triacylglycerols (Supplementary Fig. S7).

We conclude that chronic exposure to high temperature significantly decreases body fat reserves. This raises the question, whether the body fat reduction in response to elevated temperature might be a consequence of thermal stress.

### Transient thermal stress causes a lasting body fat decline in *Drosophila*

**Figure 3:**
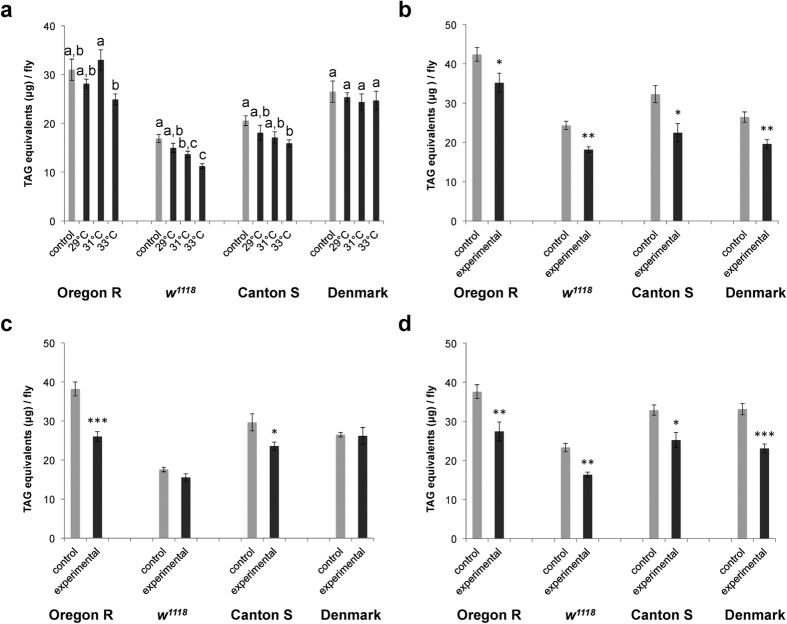
Acute thermal stress depletes body fat stores. (**a**) Exposure (24 h) to different temperatures. Data (for given population) were analysed by one-way ANOVA with Tukey’s HSD test: *P* < 0.05. Values marked with different letters are significantly different. (**b**) Heat shock (38 °C, 45 min). (**c**) Cold exposure. (4 °C, 4 h) (**d**) Cold exposure. (0 °C, 4 h). Note: body fat content was determined 24 h after acute thermal stress exposure. Data were analysed by the two-tailed Student’s *t*-test. *P < 0.05, **P < 0.01, ***P < 0.001. For global statistical analyses see Supplementary Tables S9 and S10.

To evaluate the effects of transient thermal stress on body fat content, we examined whether the fat content might be affected by a short-term exposure to higher temperatures. We found that 24 h exposure to temperatures above 31 °C has a significant negative impact on the fat content (Fig. 3a, Supplementary Table S9). Similarly, a single heat shock pulse (38 °C, 45 min) decreases the fat content within 24 hours (Fig. 3b, Supplementary Table S10). Interestingly, a similar significant depletion of body fat stores was also observed in flies 24 h after exposure for four hours to low non-freezing temperatures (4 °C: Fig. 3c; 0 °C: Fig. 3d; Supplementary Table S10). These results show that a thermal stress can significantly decrease *Drosophila* fat reserves.

**Figure 4:**
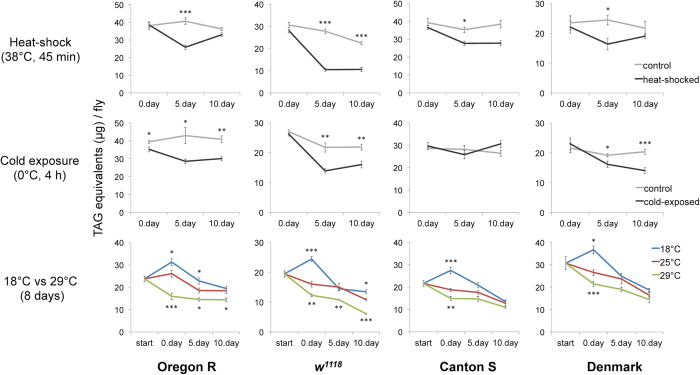
Thermal stress has long-term negative impact on body fat reserves. Day 0 represents the value measured immediately after the treatment. Data were analysed by the two-tailed Student’s *t*-test. *P < 0.05, **P < 0.01, ***P < 0.001. Error bars represent standard errors of the mean. For global statistical analyses see Supplementary Tables S12, S13 and S14.

**Figure 5:**
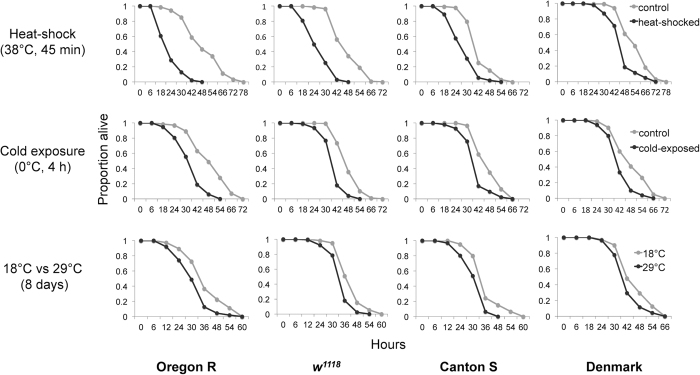
Exposure to thermal stress decreases starvation resistance. Starvation resistance was measured five days after exposure to the thermal stress. For statistical analyses see Supplementary Table S15.

Decrease and recovery of body fat levels are homeostatically regulated, such as in response to alternating fasting and feeding cycles. Flies exposed to food deprivation for 24 h lose more than 50% of their body fat stores, but re-feeding for five days enables full recovery of their fat reserves (Supplementary Fig. S8, Supplementary Table S11). To find out how quickly homeostatic regulation allows recovery of body fat storage after thermal stress, we examined the fat content of flies five and ten days after stress exposure. Surprisingly, heat-shock, cold exposure, and high temperature have a negative effect upon the fat reserves that is still detectable even five days after stress exposure (Fig. 4, Supplementary Tables S12–14). Consistent with these results, flies exposed to thermal stress are significantly less starvation resistant when examined five days after stress exposure (Fig. 5, Supplementary Table S15). This suggests that the exposure to thermal stress makes flies more vulnerable to food deprivation and might thus significantly alter their fitness in a natural environment that is characterized by varying food availability.

### The heat-shock response protects from thermal stress-induced body fat decline

**Figure 6:**
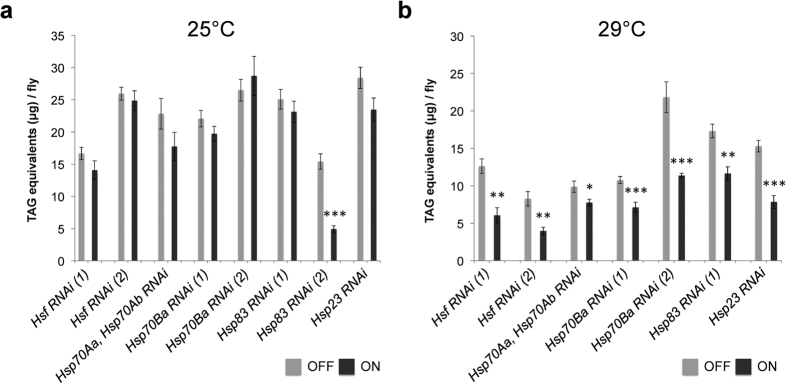
Effect of manipulation of the heat-shock response genes on the fat content. (**a**) Down-regulation of different heat-shock response genes by *da*-GS at 25 °C. (**b**) Down-regulation of different heat-shock response genes by *da*-GS at 29 °C. Note that overexpression of *Hsp83 RNAi* (2) was lethal at 29 °C. ON – with mifepristone; OFF – with solvent. For details of experimental procedure see Material and Methods. All data were analysed by the two-tailed Student’s *t*-test. *P < 0.05, **P < 0.01, ***P < 0.001. Error bars represent standard errors of the mean.

In response to thermal stress, cells induce the production of heat-shock proteins (Hsps), which prevent nonspecific aggregation of proteins and re-establish biological functionality of denatured proteins (reviewed in refs 10,11). In *Drosophila*, transcription of different *Hsp* genes in response to stress is activated by a single Heat shock transcription factor (Hsf)^24^. Among the major *Hsps* that are significantly up-regulated by both cold and heat exposure are *Hsp83, Hsp70s* and *Hsp23*^25^,^26^,^27^. To examine whether body fat storage depends on the heat-shock response machinery, we ubiquitously down-regulated the heat shock factor (*Hsf*) and stress-inducible heat shock proteins Hsp70, Hsp83 and Hsp23 by conditional RNA interference *in vivo*. Down-regulation of these major components of the *Drosophila* heat-shock response has little effect on the body fat content at normal ambient temperature (25 °C; Fig. 6a). However, at high ambient temperature (29 °C), impairment of the majority of the tested heat shock genes leads to significant decline in the lipid reserves (Fig. 6b). Thus, compromised heat-shock response sensitizes the body fat content to thermal stress. This indicates that the observed decrease in the fat content could be caused by increased cellular stress and damage, which might eventually result in the apoptotic death of fat body cells.

### Thermal stress induces apoptosis in the fat body

**Figure 7:**
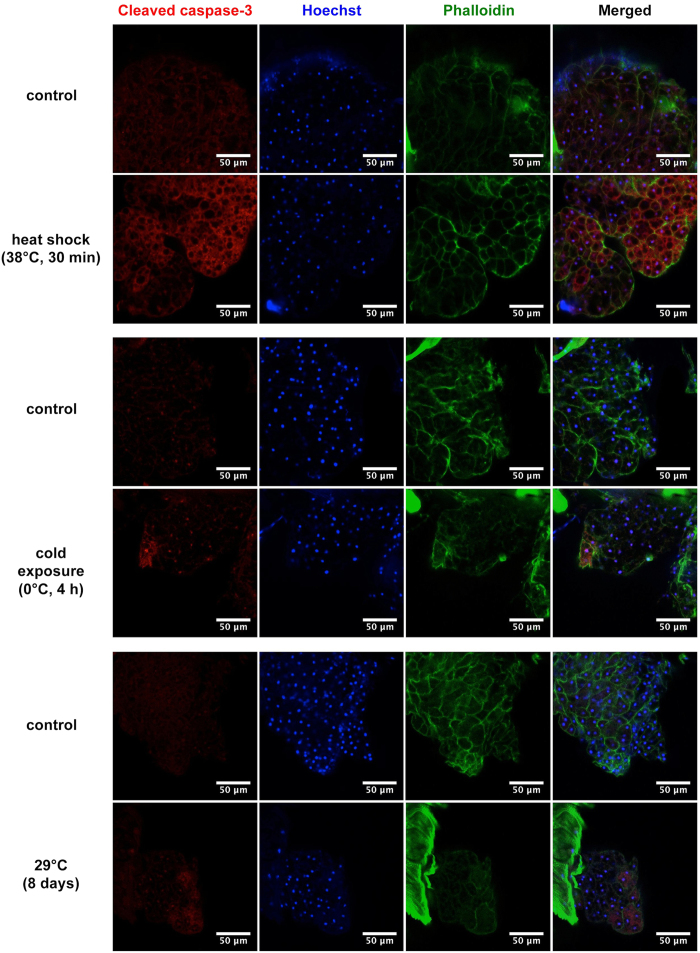
Detection of apoptosis in the fat body after thermal stress. Apoptotic cells are stained with cleaved caspase-3 antibody, DNA with Hoechst and F-actin with Phalloidin.

**Figure 8:**
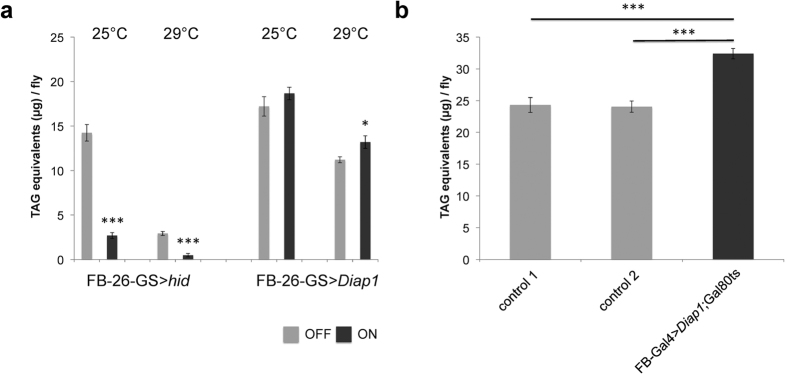
Effect of manipulation of the apoptosis-related genes on the fat content. (**a**) Up-regulation of *hid* and *Diap1* by FBI-26-GS. ON – with mifepristone; OFF – with solvent. (**b**) Up-regulation of *Diap1* by TARGET (29 °C); as controls, progeny from crosses between *w*^*1118*^ and ts-FB-Gal4 (control 1) and between *w*^*1118*^ and *UAS-Diap1* (control 2) were used. For details of experimental procedure see Material and Methods. All data were analysed by the two-tailed Student’s *t*-test. *P < 0.05, **P < 0.01, ***P < 0.001. Error bars represent standard errors of the mean.

It has been demonstrated that both heat and cold shock induce apoptosis in the *Drosophila* flight muscles^28^. To find out whether apoptosis occurs also in the adult fat body under normal and under thermal stress conditions, we performed immunohistochemistry using the cleaved caspase-3 antibody, which is a marker of the initiator caspase DRONC activity in *Drosophila*^29^. We did not observe apoptosis in the fat body at 25 °C, but we detected apoptotic fat body cells after heat-shock (38 °C, 45 min) and prolonged exposure to 29 °C (8 days), as well as after exposure to cold (0 °C, 4 h) (Fig. 7). This suggests that apoptotic removal of damaged fat storage cells might cause *Drosophila* body fat decline in response to thermal stress. To test this hypothesis, we induced apoptosis specifically in the fat body by over-expression of the pro-apoptotic gene *hid*^30^. The over-expression of *hid* significantly decreased the body fat content at both 25 °C and 29 °C ambient temperature (Fig. 8a). Conversely, we blocked apoptosis by over-expression of the anti-apoptotic gene *Diap1*^31^ using two independent fat body-specific gene expression systems (for details see Material and Methods). Consistent with the lack of apoptosis in the fly fat body at 25 °C the over-expression of *Diap1* has little effect on body fat content at this temperature (Fig. 8a). At elevated ambient temperature (29 °C), however, the inhibition of apoptosis in the fat body significantly attenuates the temperature-induced decrease in the fat content (Fig. 8a,b). These data suggest that the apoptosis of fat body cells at least partially contributes to the observed depletion of body fat reserves induced by thermal stress and the compromised recovery of body fat content after stress exposure.

## Discussion

### Inverse relation between temperature and organismal fat reserves

In this study, we have examined the effect of temperature and thermal stress on the energy reserves in *D. melanogaster*. We found that lipid reserves are sensitive to ambient thermal changes. Interestingly, glycogen levels are rather stable. This might be explained by the fact that glycogen is a main source of the hemolymph sugar trehalose, which fuels insect flight^20^. In *Drosophila*, glycogen is actually the sole reserve, which is utilized during flight^32^. Therefore, glycogen level stability might be vital for sustaining flight capability. Fat reserves significantly decreased at higher temperature in all examined populations, despite constant availability of a food resource. In addition, we also observed that flies could increase their fat reserves at lower temperatures. One possible explanation is that at higher temperatures the energy demands (higher metabolic rate) exceed the energy acquisition, with consequent energy deficit being covered by utilization of lipid reserves. Conversely, energy intake at lower temperatures might surpass energy expenditure, allowing accumulation of body fat reserves. Notably, it has also been observed that the relation between temperature and the rate of energy acquisition and expenditure might not be scaled. For example, Rall *et al*.^33^ have found that ingestion efficiencies (ingestion/metabolism) of different arthropods (beetles and spiders) were reduced at higher temperature. Similarly, Lemoine and Burkepile^34^ have revealed that the metabolic rate of the urchin *Lytechinus variegatus* increased with temperature more rapidly than consumption. Whether such a mismatch between metabolism and consumption exists also in *D. melanogaster* is currently under investigation.

In the long term, elevated ambient temperature might be stressful for organisms. According to the concept of energy-limited tolerance to stress^13^, relatively more energy is devoted to somatic maintenance under non-optimal conditions, whereas the accumulation of energy reserves is reduced or the energy stores even become depleted. Exposure to non-optimal temperatures might impose additional costs due to the energetically demanding production of heat shock proteins^35^, for example. Similarly, refolding and degradation of damaged proteins, as well as synthesis of new proteins that replace irreversibly damaged ones are all costly processes that might considerably increase the energy requirements of the cell^36^,^37^. A key feature of the cellular stress response is the inhibition of growth and proliferation, which allows redirection of the energy towards macromolecular maintenance and repair (reviewed in ref. 9). Altogether, this implies that elevated ambient temperature might significantly affect the demand, the utilization and the allocation of energy^37^, which may ultimately reduce the organismal energy reserves.

### Thermal stress decreases body fat reserves and starvation resistance

Apart from studying how longer exposure to temperatures within thermal tolerance limits of *Drosophila* influences energy reserves, we also examined the effect of a shorter exposure to stressful temperatures on body fat content. We found that transient exposure to both high and low temperatures reduces fly body fat reserves. Importantly, we did not detect any significant differences in the body fat content immediately after the treatment. Similarly, a 24-hour exposure to temperatures above 31 °C or a short term exposure to sub-lethal high temperature, which causes partial and transient heat knockdown of flies, does not have an instantaneous impact on the body fat content. In all cases the depletion of body fat reserves was detectable only after the exposure to the non-lethal stressful conditions.

Our findings are consistent with previous studies that profiled *Drosophila* metabolites after thermal stress exposure. Malmendal *et al*.^38^ have detected a decrease of several key metabolites of energy homeostasis, such as fatty acid-like metabolites, glycogen and glucose, during the recovery phase (8 hours) following exposure to heat stress. Interestingly, these metabolites did not recover to normal values within eight hours after the heat stress. The authors argue that the observed metabolite reduction might be caused by a metabolic rate increase during heat stress^38^. A similar, substantial decrease of *Drosophila* fatty acid-like metabolites immediately after rapid cold hardening or one day after cold shock has been reported by Overgaard *et al*.^39^. Opposite to heat stress, cold exposure caused an increase in glucose and trehalose levels in this study. Notably, whereas sugars returned to control values within three days, the level of fatty acid-like metabolites remained lower compared to controls even after this period^39^. In this context, it would be interesting to examine the food intake immediately after heat-shock or cold exposure, since recovery from heat knockdown or chill-coma might affect feeding behaviour and thus might cause a utilization of glycogen/fat reserves during this fasting period. However, since flies starved for one day are able to fully replenish their body fat reserves within five days (Supplementary Fig. S8) (interestingly, starvation-exposed flies have even higher fat content than controls after five days, see Supplementary Table S11), any possible short-term food deprivation cannot explain the long-lasting depletion of body fat reserves in response to transient thermal stress.

It is well documented that mild heat or cold stress increases resistance to severe stress in *Drosophila* (e.g. refs 40,41). On the other hand, it has also been observed that thermal stress decreases fecundity and the number of offspring produced and thus might significantly affect fitness (e.g. refs 40,42). Since fat reserves play an important role in oogenesis (reviewed in ref. 20), it would be interesting to examine whether the documented reduction in progeny number after thermal stress might be related to stress-associated depletion of body fat content or if it is rather a consequence of stress-induced apoptosis of egg-chambers (e.g. ref. 43). In agreement with the observed decrease in body fat content, we found that thermally stressed flies have reduced starvation resistance even five days after the end of stress exposure. Consistently, reduced starvation resistance after cold stress has been reported earlier^41^. Since food shortage is a strong selection agent in natural populations (e.g. refs 16,17), any transient exposure to thermal stress might also significantly affect individual survival.

### Stress, aging and apoptosis

We found that conditional ubiquitous down-regulation of different components of the heat-shock response machinery significantly reduces the body fat content at elevated ambient temperature. These results are consistent with previous findings that manipulation of Hsf, Hsp83, and Hsp70 influences sensitivity to thermal stress^24^,^44^,^45^. In agreement with the role of Hsps in the mitigation of the temperature-induced damages, our results suggest that temperature-induced depletion of fat reserves might be directly related to increased cellular stress and damage caused by exposure to stressful conditions.

If the protective mechanisms in a stress-exposed cell are not able to prevent accumulation of macromolecular damages, the affected cell activates the apoptotic suicide pathway (reviewed in ref. 12). Several studies have found that both heat and cold stress induce apoptosis in insect tissues (e.g. refs 28,46), including in the fat body of the flesh fly *Sarcophaga crassipalpis*^47^. Consistently, our immunohistochemistry analysis showed that thermal stress induces apoptosis of *Drosophila* adipocytes. In addition, we revealed that the activation or inhibition of apoptosis specifically in the fly fat body causes a significant reduction or increase in the amount of body fat, respectively. These results indicate that the observed depletion of body fat reserves in response to thermal stress is at least partially caused by apoptosis of adipocytes in the fly fat body. The irreversible fat body cell loss due to apoptosis may also explain the observed compromised recovery of body fat reserves after stress.

Importantly, Zheng *et al*.^48^ have shown that *Drosophila* aging is coupled with progressive apoptosis in muscle and fat body cells. In agreement with this study, our analyses of age-related changes in the body fat content revealed a progressive age-dependent decline in body fat reserves. This is in line with a reduction of the abdominal subcuticular adipose tissue volume in older compared to young flies (Fig. S8). These results agree well with previous findings that the starvation resistance of *Drosophila* males declines with age^23^. The fact that the age-related decline in fat reserves is faster at higher temperature (Fig. 2) parallels the inverse relation between ambient temperature and lifespan (e.g. ref. 49). As the fly ages, damages to macromolecules progressively accumulate, which causes cellular defects, aging and ultimately death^50^. Since the accumulation of damages is accelerated by elevated temperature (e.g. ref. 51) or by environmental stress in general (reviewed in ref. 9), it is reasonable to presume that the common mechanism underlying both age-related and stress-induced depletion of body fat reserves might be apoptotic cell death of adipocytes triggered by damage accumulation.

Our results show that elevated temperature and thermal stress deplete organismal fat reserves. Importantly, thermal stress has a lasting effect on the body fat storage, which significantly impairs the ability of flies to survive starvation. Subsequent functional analyses revealed that the temperature-dependent decrease in body fat reserves is at least in part caused by apoptosis of fat body cells possibly triggered by stress-induced cellular damage. In view of the rapid climate change, sustained depletion of body fat reserves in response to ambient temperature fluctuations might represent an important physiological phenomenon affecting the fitness of ectothermic organisms.

## Materials and Methods

### Fly stocks and maintenance

*D. melanogaster* stocks used in this study: Oregon R, Canton S, *w*^*1118*^ (VDRC 60000), recently collected wild population of *D. melanogaster* from Denmark (for further details see ref. 22). Additional stocks used in this study: *da-*GS^52^; *FBI-26-*GS^53^; ts-FB-Gal4^54^; *Lpp-*Gal4^55^; UAS-StingerII^56^; UAS*-hid*; UAS*-Diap1; Hsf RNAi; Hsp83 RNAi; Hsp70Aa; Hsp70Ab RNAi; Hsp70Ba RNAi; Hsp23 RNAi* (for details see Supplementary Table S16).

Unless stated otherwise, all experiments were conducted on standard *Drosophila* medium (5.43 g agar, 15.65 g yeast, 8.7 g soy flour, 69.57 g maize flour, 19.13 g beet syrup, 69.57 g malt, 5.43 ml propionic acid and 1.3 g methyl 4-hydroxybenzoate per 1 l of medium).

All experiments were performed on male flies. If not specified otherwise, flies developed at 25 °C (12h:12h L:D cycle; 60–70% humidity) and medium egg density (approx. 150 eggs per 68 ml vial). Males were collected within 24 h after eclosion and reared on standard medium (approx. 50 males per vial). Flies were transferred daily to fresh medium.

Conditional *in vivo* manipulation of individual gene expressions was achieved either by the ubiquitous *daughterless-*GeneSwitch (*da-*GS)^52^ or by the fat-body-specific FBI-26-Geneswitch (FBI-26-GS)^53^. Additionally, we also used the temperature-inducible TARGET system^57^ to overexpress *UAS-Diap1* in the fat body (ts-FB-Gal4^54^). To induce gene switch (*da-*GS and FBI-26-GS), four-day-old males that were raised at 25 °C (12:12 L:D cycle; 60–70% humidity), were placed on standard medium containing either 200 μM RU486 (mifepristone; Sigma M8046; stock solution 20 mM in ethanol; ON condition) or solvent (OFF condition) and were kept for 8 days either at 25 °C or 29 °C (12:12 L:D cycle; 60–70% humidity) with food exchange every other day; mifepristone itself did not have a significant effect on the body fat content (Supplementary Fig. S9). To induce the temperature-inducible TARGET system, flies carrying the ts-FB-Gal4 driver developed and were kept at 18 °C (OFF conditions) (12:12 L:D; 60–70% humidity). After four days, these flies were transferred to 29 °C (ON conditions) (12:12 L:D; 60–70% humidity) for 8 days with food exchange every other day. As controls, we used progeny from crosses between *w*^*1118*^ and ts-FB-Gal4 (control 1) and between *w*^*1118*^ and *UAS-Diap1* (control 2).

### Thermal experiments

To examine the effect of temperature on the body fat content, we used a full factorial experimental design (Supplementary Fig. S1). In detail, parental flies were allowed to lay eggs for 12 h at 25 °C in 68 ml vials. Vials (150 eggs per vial) were randomly distributed to three temperatures: 18 °C, 25 °C and 29 °C (12:12 L:D; 60–70% humidity). After eclosion, males were kept at the ‘initial’ temperature (which was identical to the temperature experienced during development) for 4 days. This 4 days period was used in order to ensure that the larval fat body is completely removed^58^. Afterwards, flies were randomly transferred into 18 °C, 25 °C and 29 °C (12:12 L:D; 60–70% humidity) (‘adulthood’ temperature). After 8 days, random samples were collected for the lipid and glycogen determination. The whole experiment was performed also on high protein medium (see Supplementary Material and Methods).

### Age-related changes in the fat content

To determine age-dependent changes in the body fat content at different temperatures, eggs were collected as previously described and randomly distributed into 18 °C, 25 °C and 29 °C (12:12 L:D; 60–70% humidity). After eclosion, males were collected and kept at given temperatures for 30 days with daily food exchange. Random samples of flies for the lipid determination were collected within 24 h after eclosion and on day 5, 10, 15, 20 and 30. Since based on our previous experience median lifespan of *Drosophila* males at 29 °C is approximately 32–36 days, the 30-days period thus represents a significant part of the lifespan at 29 °C before the onset of substantial mortality.

### Thermal stress induction

To determine the effect of heat-shock on the body fat content, four-day-old males (approx. 30 males per replicate; at least two replicates per genotype and treatment) were placed into glass tubes and exposed either to 25 °C (controls) or to 38 °C for 45 minutes in the water-bath. To examine the changes in the body fat content at high and low temperatures, four-day-old males (approx. 30 males per replicate; at least two replicates per genotype and treatment) kept in vials with standard medium were randomly distributed to 25 °C (control), 29 °C, 31 °C and 33 °C for 24 hours or in the case of the low temperatures to either 4 °C or 0 °C (ice-water-bath) for 4 hours. After all treatments, flies were returned to 25 °C on standard medium, which was changed daily. None of the stressful treatments caused an immediate or delayed (within 24 hours) increase in mortality. Random samples of flies for the lipid determination were collected immediately before and after the treatment and 24 hours after the exposures. To examine the long-term effect of heat and cold exposures, flies were collected immediately after the treatment and 5 and 10 days later.

### Lipid and glycogen determination

Flies were collected and homogenates for all metabolic measurements were prepared as described in Gáliková *et al*.^21^. Lipid determination by coupled colorimetric assay was performed as described by Hildebrandt *et al*.^59^. Glycogen measurements were done as described in Gáliková *et al*.^21^. For each genotype and experimental treatment, four to eight replicates of five males each were examined. Fat content is expressed as μg triacylglycerol (TAG) equivalents/fly. Similarly glycogen content is expressed as μg glycogen/fly.

### Starvation resistance assay

Four-day-old male flies (three replicates per genotype and treatment; approx. 50 males per replicate) were exposed to either heat shock (38 °C for 45 min), or to cold (0 °C for 4 h) or to 18 °C versus 29 °C (for 8 days). After the treatment, flies were allowed to recover for five days at 25 °C with daily food exchange. Subsequently, flies were transferred to 1% agarose (in water), and starvation survival was monitored at 25 °C (12:12 L:D; 60–70% humidity) by counting the number of dead flies every 6–12 h until the death of the last fly.

### Immunohistochemistry

Male flies (*w*^*1118*^) exposed to heat-shock (38 °C, 45 min), cold (0 °C, 4 h) or to 29 °C (8 days) were anesthetized on ice 2 h after the exposure, and their abdominal carcass with the subcuticular fat body was dissected in chilled 1 x PBS (phosphate-buffered saline) and fixed for 35 min in 4% paraformaldehyde at room temperature. After three washes with PBST (PBS with 0.3% Triton X-100), samples were blocked 20 min with PBST-S (2% bovine serum albumin (Biomol) in PBST) and 50 min with PBST-S-S (2% sheep serum (Sigma-Aldrich) in PBST-S). Samples were incubated with rabbit cleaved caspase-3 (Asp 175) antibody (1:200; New England Biolabs, 9661) overnight at 4 °C. After five washes in PBST, samples were blocked 15 min with PBST-S and 50 min with PBST-S-S. Secondary antibody used was anti-rabbit Alexa Fluor 568 (1:100; Invitrogen, A-11036). Our test of secondary antibody revealed that this antibody bound non-specifically to the oenocytes. DNA was stained with Hoechst 33342 (1:100; PromoKine, PK-CA-707-30018), and to visualize F-actin Alexa Fluor 488 Phalloidin (1:100; Invitrogen A-12379) was used. After overnight incubation at 4 °C and 2.5 h incubation at 25 °C, samples were washed five times in PBST. All samples were mounted in Mowiol (Mowiol 4–88; Calbiochem, 475904) mounting medium and viewed on a Zeiss LSM-780 confocal microscope. Images were processed using ImageJ v1.50e.

### Statistical analyses

The effects of different factors (population, initial temperature, adulthood temperature, starvation as fixed effects) and their interactions on variation in the fat and glycogen content and on the relative changes in the fat content were analysed using either three-way or two-way analysis of variance (ANOVA). One-way ANOVA, followed by Tukey’s HSD (honestly significant difference) post-hoc tests with α = 0.05 was used to test the effect of a single fixed factor on the fat or glycogen content. Pairwise comparisons of means were conducted using the Student’s *t*-test.

To compare the age-related changes in the fat content at three temperatures, we fitted first-, second- and third-degree polynomial functions to our data using JMP v.12.2 software. Based on the Akaike information criterion^60^, our data were best described in all four populations by the first-degree polynomial (linear) function. Slopes of the fitted lines were compared by the parallelism *F*-test.

Pairwise differences in starvation survival were tested using the log-rank test and the Wilcoxon rank sum test.

All analyses were performed with JMP v.12.2 (SAS, Raleigh, NC, USA) and PAST 3.11^61^.

## Additional Information

**How to cite this article**: Klepsatel, P. *et al*. Thermal stress depletes energy reserves in *Drosophila. Sci. Rep.* **6**, 33667; doi: 10.1038/srep33667 (2016).

## Supplementary Material

Supplementary Information
