# Supplementary material for: Thermal stress depletes energy reserves in Drosophila
Source: Sci Rep. 2016 Sep 19;6:33667. doi: 10.1038/srep33667 (PMC5027548; doi:10.1038/srep33667)
Supplement: Supplementary Information [file srep33667-s1.pdf]

Supplementary Information

Thermal stress depletes energy reserves in *Drosophila*

Peter Klepsatel*, Martina Gáliková, Yanjun Xu and Ronald P. Kühnlein

Supplementary Material and Methods

High protein medium

Except of the standard diet (see Material and Methods), we used also high protein medium with four times increased yeast content (62.6 g yeast per 1 l of medium) in comparison to the standard medium. The amounts of other components remained unchanged. This medium was not used during development of flies but only after eclosion, during adulthood.

Thin layer chromatography

Thin layer chromatography (TLC) was conducted as described in Gáliková *et al.*[1]. After eclosion, w^{1118} male flies were kept at 25°C; after 4 days, they were randomly transferred either into 18°C or 29°C (12:12 L:D; 60-70% humidity) for 8 days; at the end of this period samples for the TLC analysis were collected (5 flies per replicate; 3 biological replicates per temperature).

Supplementary Figures and Tables

Supplementary Figures

Supplementary Figure S1. Full factorial experimental design with three 'initial' and three 'adulthood' temperatures.

Supplementary Figure S2. Effect of temperature on the energy reserves of male flies on the high protein medium. Data (for given population and initial temperature) were analysed by one-way ANOVA with Tukey's HSD test: $P < 0.05$. Error bars represent standard errors of the mean. For global statistical analyses see Supplementary Tables S2 and S4.

Supplementary Figure S3. Effect of initial temperature on the relative changes in the body fat content at three adulthood temperatures measured on the standard diet. All values are standardized to the starting values, i.e. values measured before exposure to different temperatures. Data (for given population and adulthood temperature) were analysed by one-way ANOVA with Tukey's HSD test: $P < 0.05$. Values marked with different letters are significantly different. Error bars represent standard errors of the mean. For global statistical analyses see Supplementary Table S5.

Supplementary Figure S4. Effect of initial temperature on the relative changes in the body fat content at three adulthood temperatures measured on the high protein diet. All values are standardized to the starting values, i.e. values measured before exposure to different temperatures. Data (for given population and adulthood temperature) were analysed by one-way ANOVA with Tukey's HSD test: $P < 0.05$. Values marked with different letters are significantly different. Error bars represent

standard errors of the mean. For global statistical analyses see Supplementary Table S6.

Supplementary Figure S5. Effect of different developmental temperatures on the body fat content at eclosion. Data (for given population) were analysed by one-way ANOVA with Tukey's HSD test: $P < 0.05$. Values marked with different letters are significantly different. Error bars represent standard errors of the mean. For global statistical analyses see Supplementary Table S7.

Supplementary Figure S6. Comparison of the subcuticular abdominal (dorsal) fat body between young (4 days old) and older (31 days old) male flies (*Lpp*-Gal4>UAS-StingerII) at 25°C. *Lpp*-Gal4 is fat body-specific Gal4-driver[2]; UAS-StingerII is a GFP reporter with nuclear localization[3].

Supplementary Figure S7. Thin layer chromatography analysis shows that high temperature decreases the amount of triacylglycerols (TAG) (FA – fatty acids; DAG – diacylglycerol; MAG – monoacylglycerol). For details see Supplementary Material and Methods.

Supplementary Figure S8. Recovery of the body fat content after 24 h starvation (1% agarose; 25°C, 12:12 L:D, 60-70% humidity). 0. day represents the value measured immediately after the starvation. Data were analysed by the two-tailed Student's *t*-test. ** $P < 0.01$, *** $P < 0.001$. Error bars represent standard errors of the mean. For global statistical analyses see Supplementary Table S11.

Supplementary Figure S9. Exposure to mifepristone (RU486) does not have significant effect on the body fat content (*w*^{1118} males; 29°C, 12:12 L:D, 60-70% humidity; exposure time: 8 days). RU 0 – only solvent (ethanol); RU 200 - 200μM RU486. Data were analysed by the two-tailed Student's *t*-test. Error bars represent standard errors of the mean.

Supplementary Figure S1.

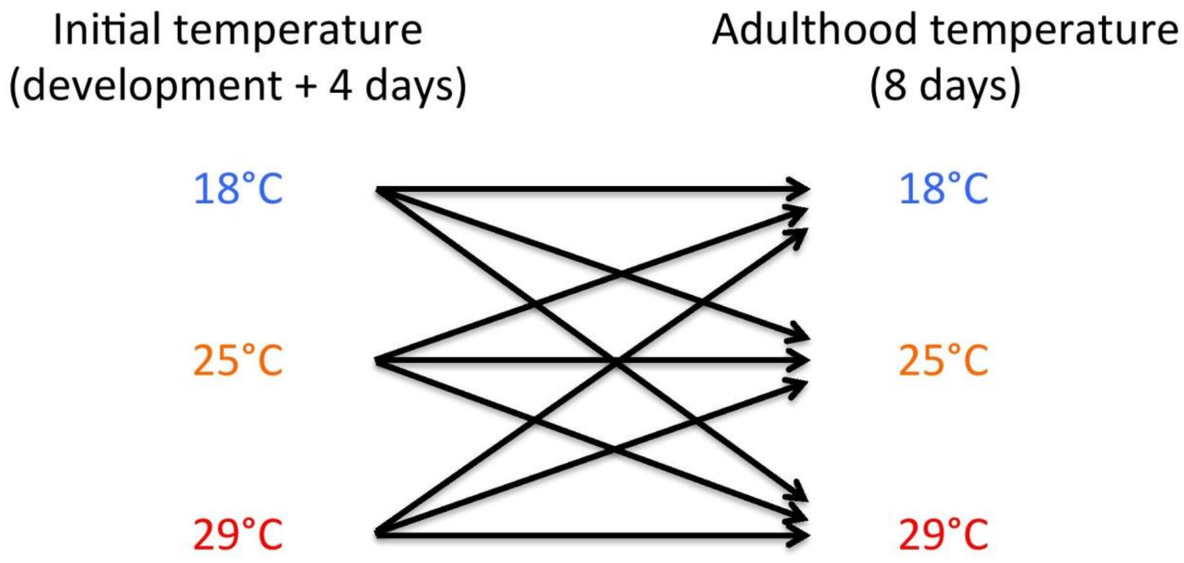

Supplementary Figure S2.

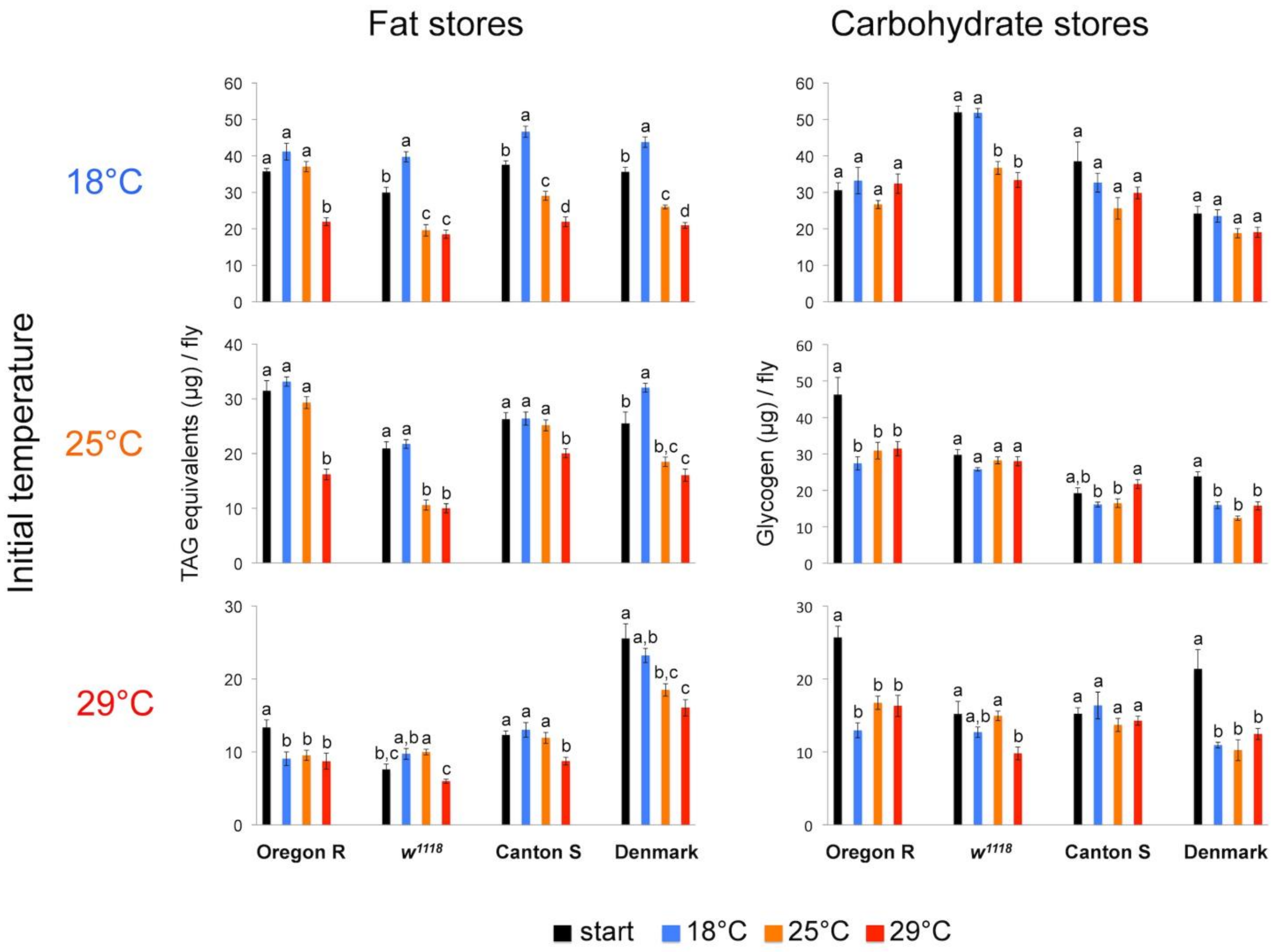

Supplementary Figure S3.

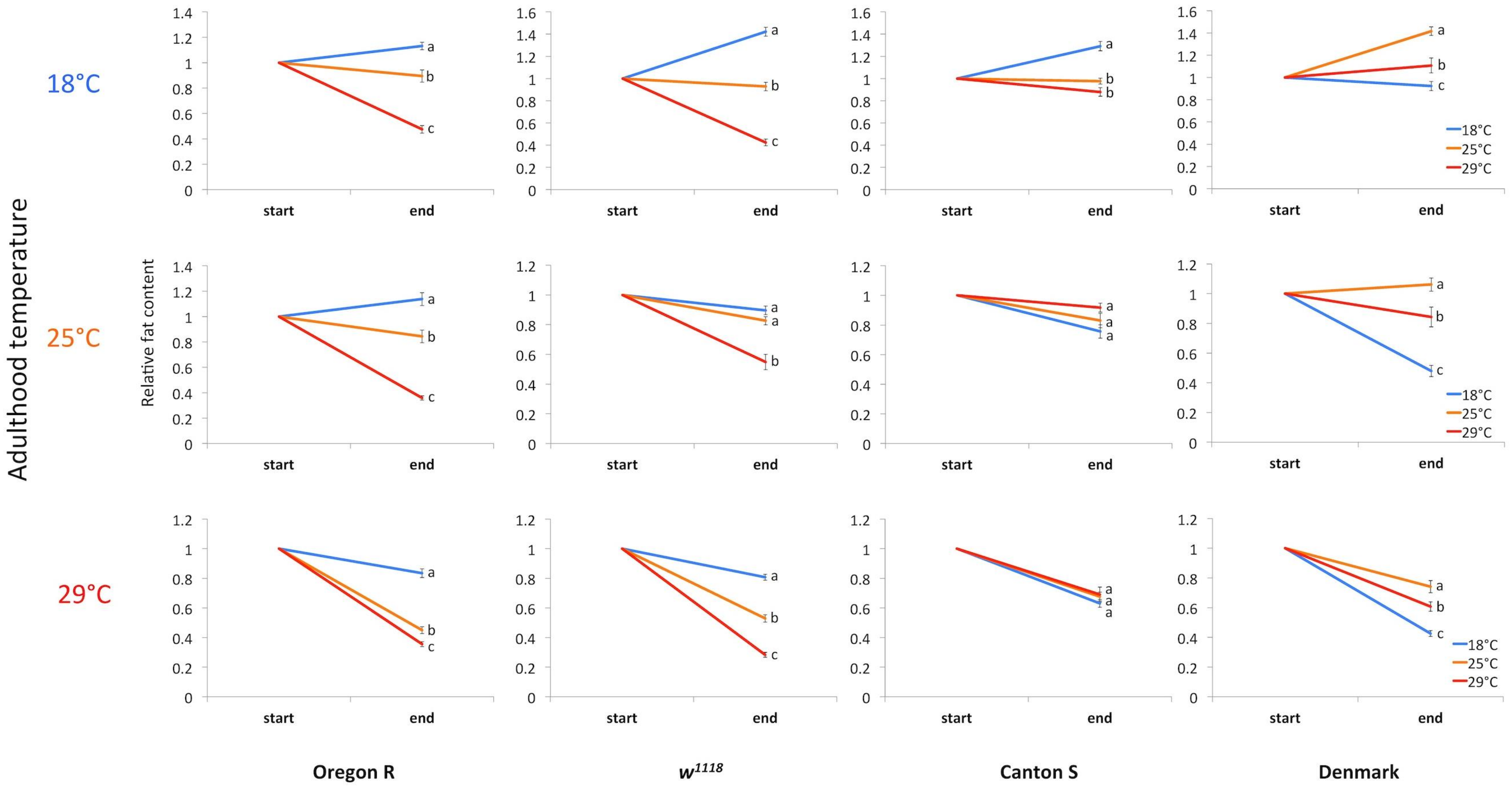

Supplementary Figure S4.

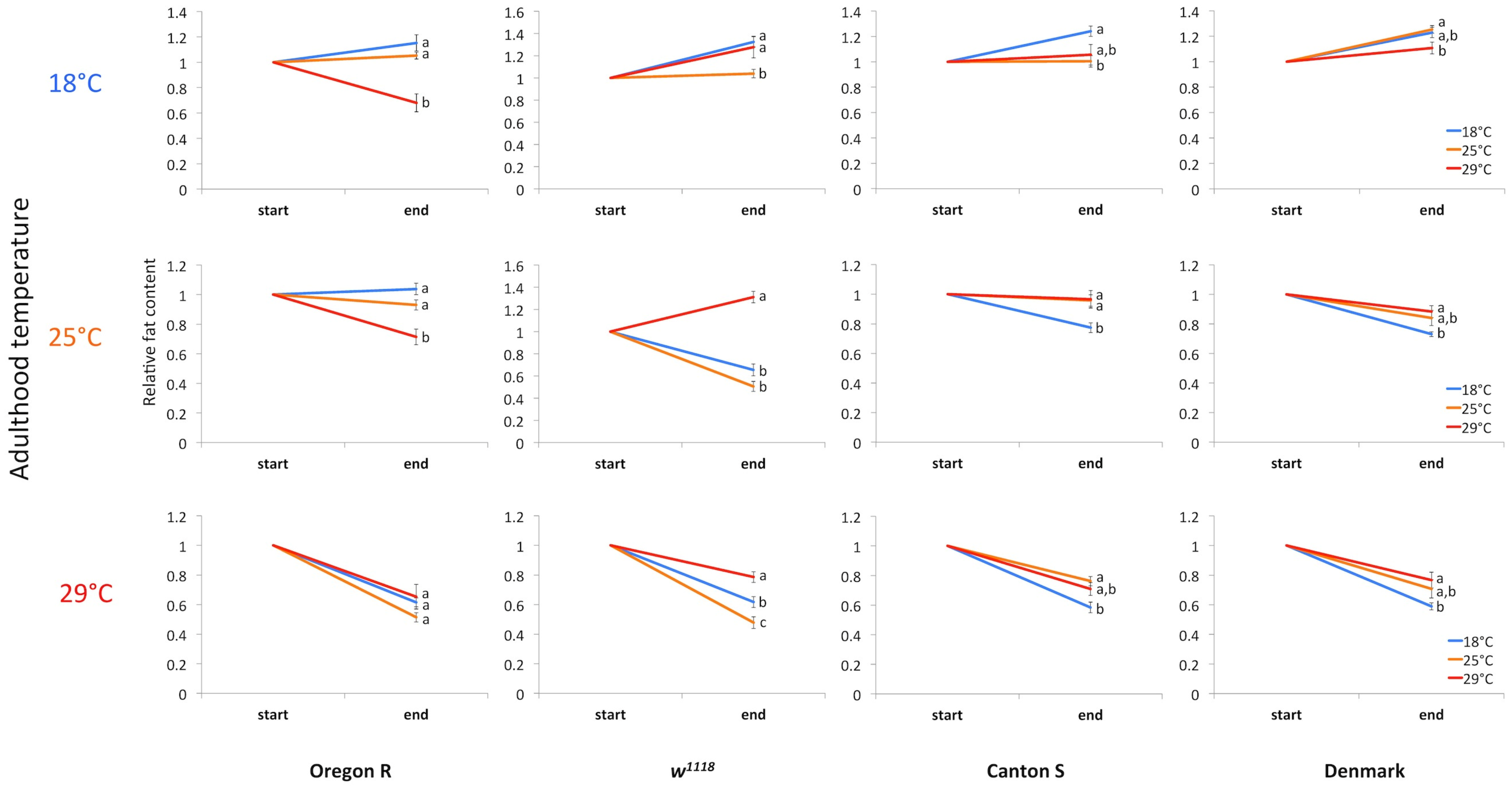

Supplementary Figure S5.

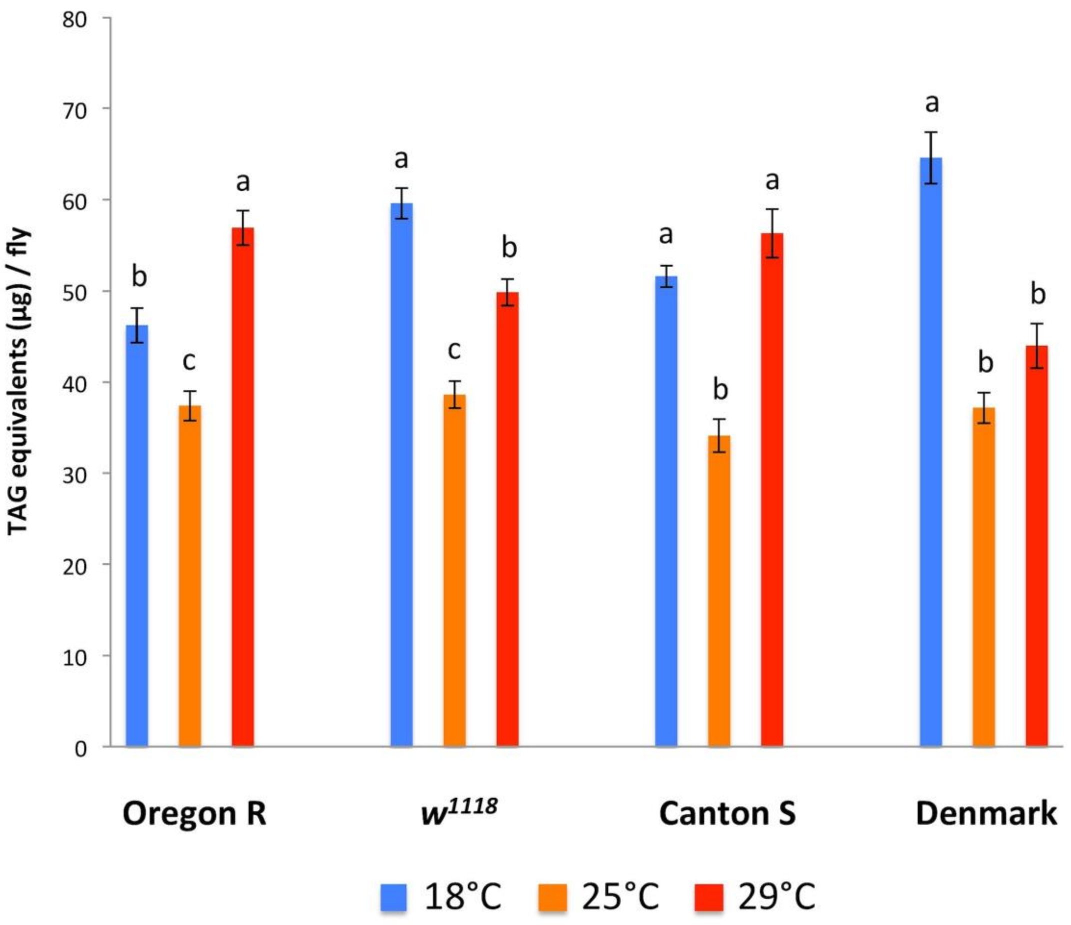

Supplementary Figure S6.

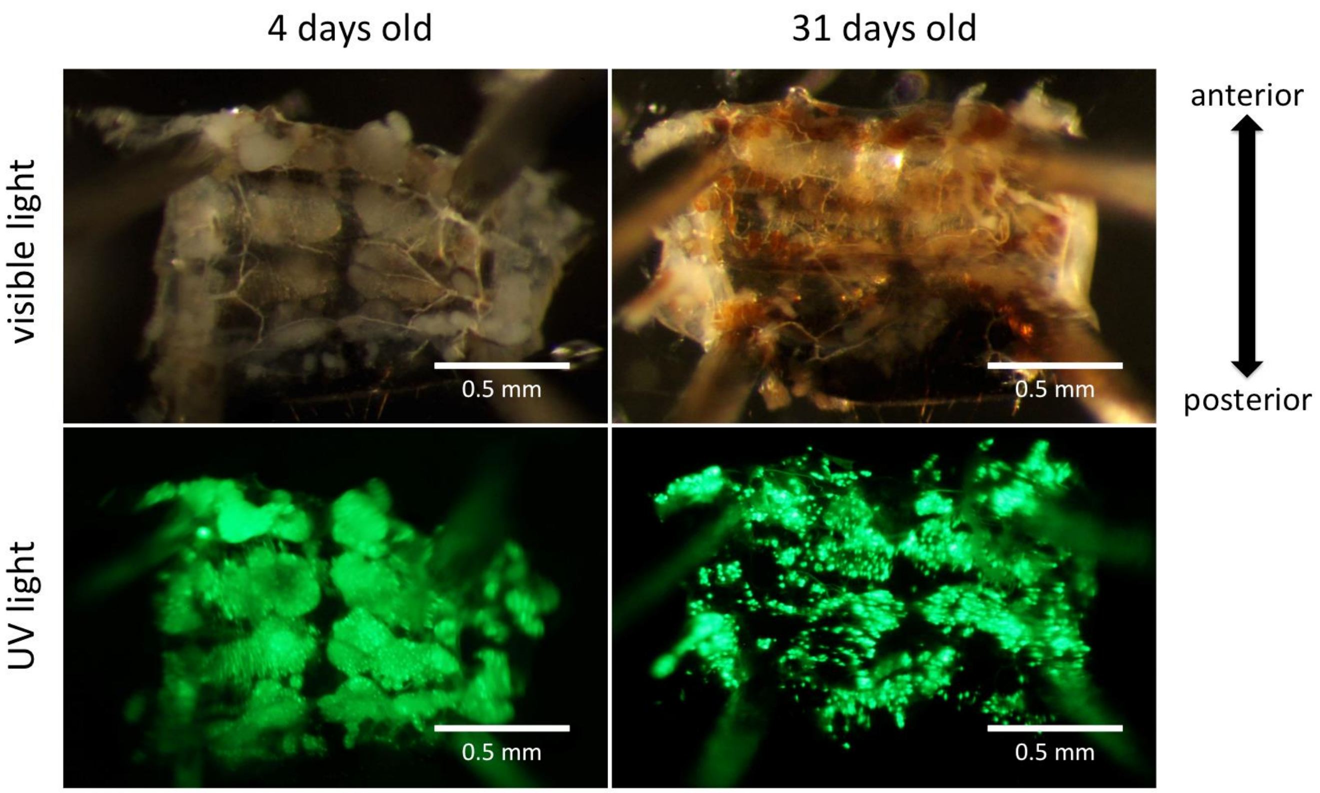

Supplementary Figure S7.

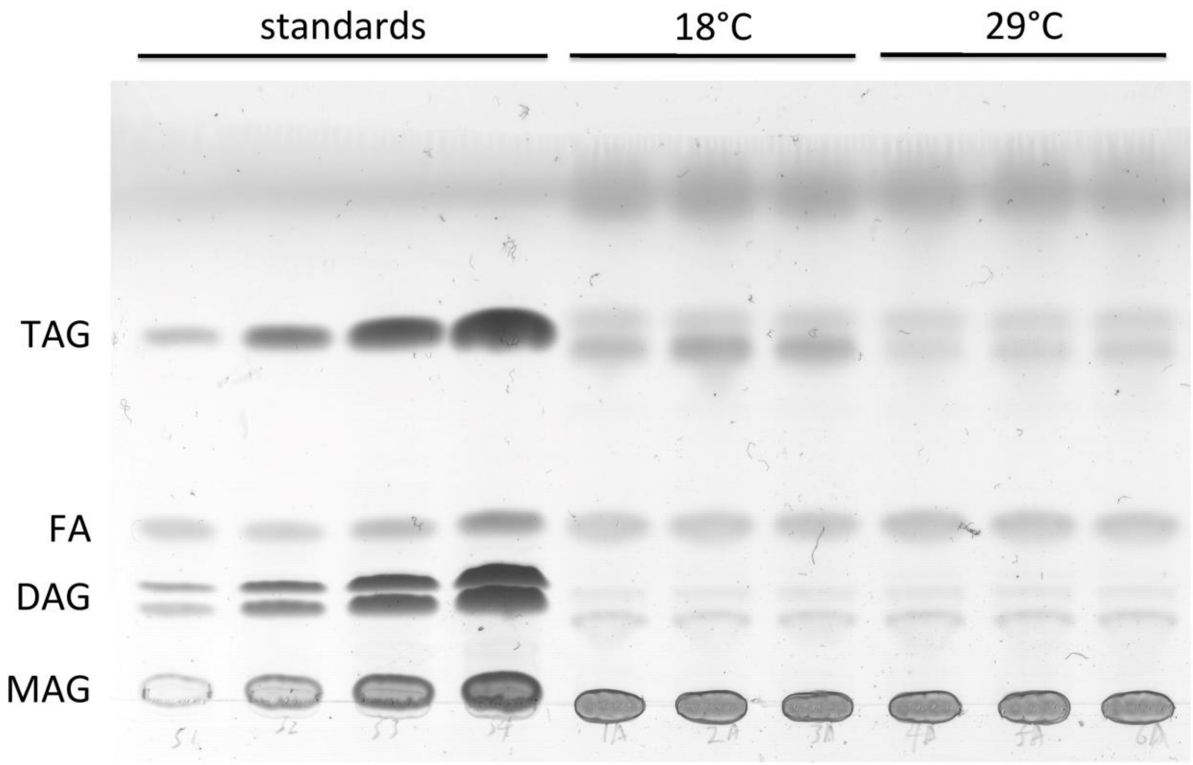

Supplementary Figure S8.

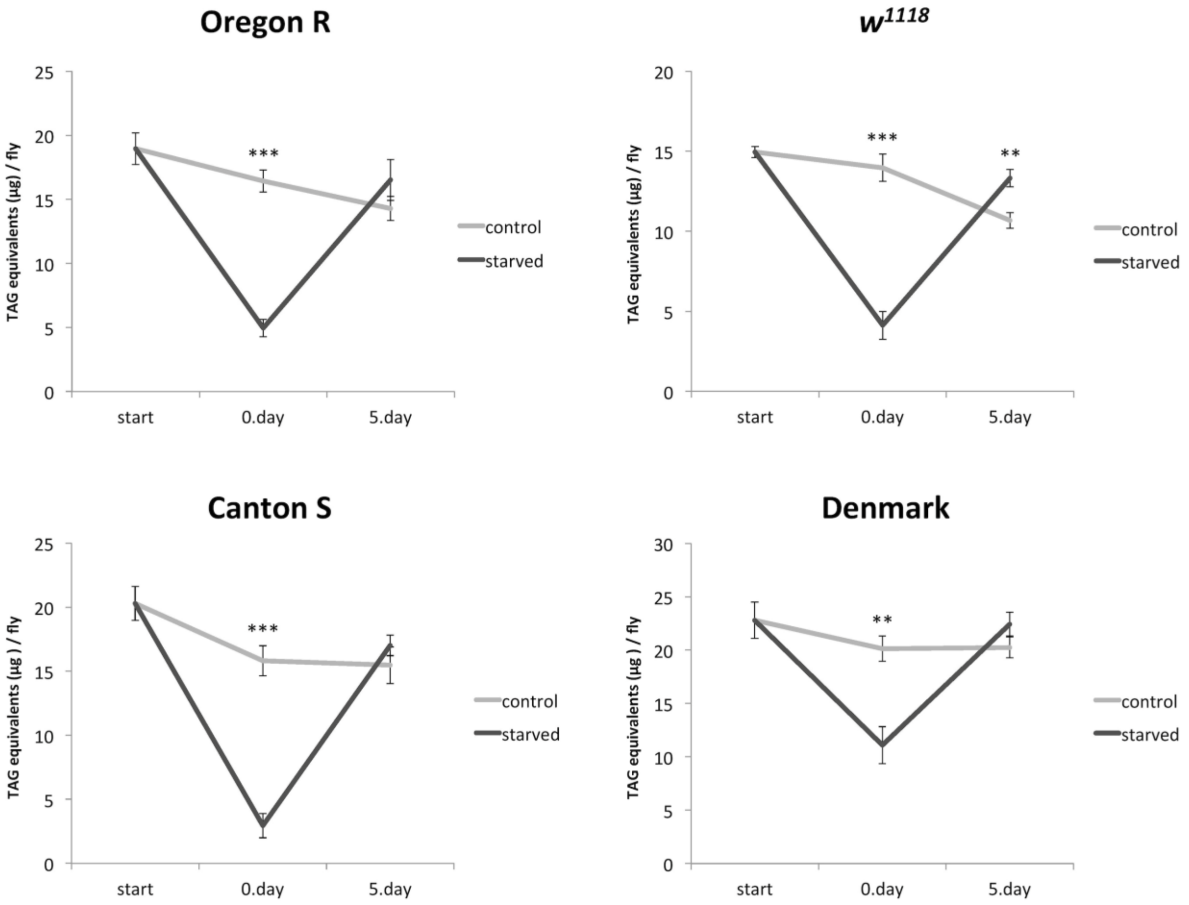

Supplementary Figure S9.

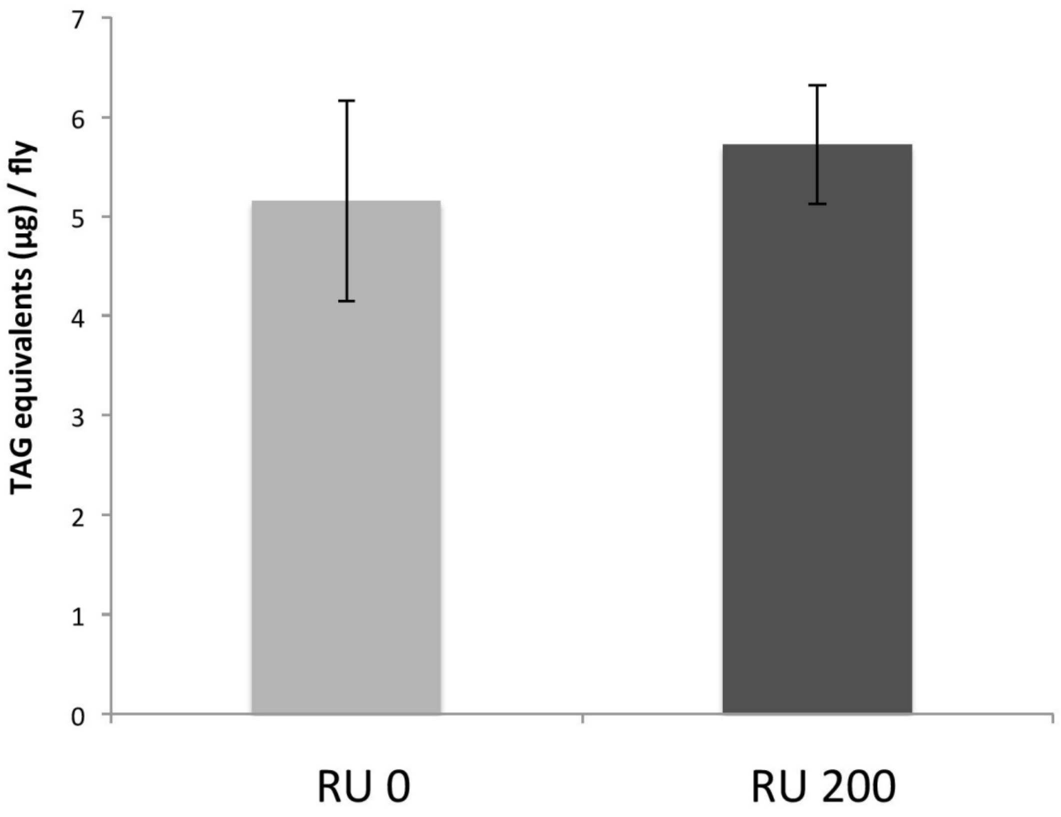

Supplementary Tables

Supplementary Table S1. Three-way analysis of variance (ANOVA) testing the effects of population, initial temperature, adulthood temperature and their interactions on the fat content (µg TAG equivalents/ fly) on the standard medium. *df* - degrees of freedom; SSQ - the sum of squares for each source of variation.

**Source of variation**	***df***	**SSQ**	***F*-ratio**	***P*-value**
Population	3	3457.0	107.2	< 0.0001
Initial temperature	2	22706.4	1056.2	< 0.0001
Adulthood temperature	2	6684.3	310.9	< 0.0001
Population × Initial temperature	6	4903.9	76.0	< 0.0001
Population × Adulthood temperature	6	568.9	8.8	< 0.0001
Initial × Adulthood temperature	4	2127.6	49.5	< 0.0001
Population × Adulthood temperature x Initial temperature	12	955.8	7.4	< 0.0001
Error	239	2569.0	-	-

Supplementary Table S2. Three-way analysis of variance (ANOVA) testing the effects of population, initial temperature, adulthood temperature and their interactions on the fat content (µg TAG equivalents/fly) on the high protein medium. *df* - degrees of freedom; SSQ - the sum of squares for each source of variation.

**Source of variation**	***df***	**SSQ**	***F*-ratio**	***P*-value**
Population	3	2651.9	88.8	< 0.0001
Initial temperature	2	15680.3	787.6	< 0.0001
Adulthood temperature	2	7591.3	381.3	< 0.0001
Population × Initial temperature	6	1740.9	29.2	< 0.0001
Population × Adulthood temperature	6	657.1	11.0	< 0.0001
Initial temperature × Adulthood temperature	4	2732.1	68.6	< 0.0001
Population × Adulthood temperature x Initial temperature	12	747.4	6.3	< 0.0001
Error	245	2438.7	-	-

Supplementary Table S3. Three-way analysis of variance (ANOVA) testing the effects of population, initial temperature, adulthood temperature and their interactions on the glycogen content (µg glycogen/ fly) on the standard medium. *df* - degrees of freedom; SSQ - the sum of squares for each source of variation.

**Source of variation**	***df***	**SSQ**	***F*-ratio**	***P*-value**
Population	3	4701.5	75.6	< 0.0001
Initial temperature	2	14858.1	358.3	< 0.0001
Adulthood temperature	2	316.6	7.6	0.0006
Population × Initial temperature	6	6094.8	49.0	< 0.0001
Population × Adulthood temperature	6	348.4	2.8	0.0119
Initial temperature × Adulthood temperature	4	1400.5	16.9	< 0.0001
Population × Adulthood temperature x Initial temperature	12	774.2	3.1	0.0004
Error	235	4872.7	-	-

Supplementary Table S4. Three-way analysis of variance (ANOVA) testing the effects of population, initial temperature, adulthood temperature and their interactions on the glycogen content (μg glycogen/ fly) on the high protein medium. *df* - degrees of freedom; SSQ - the sum of squares for each source of variation.

**Source of variation**	***df***	**SSQ**	***F*-ratio**	***P*-value**
Population	3	5479.5	90.5	< 0.0001
Initial temperature	2	13016.2	322.4	< 0.0001
Adulthood temperature	2	255.2	6.3	0.0021
Population × Initial temperature	6	3087.7	25.5	< 0.0001
Population × Adulthood temperature	6	538.0	4.4	0.0003
Initial temperature × Adulthood temperature	4	1124.7	13.9	< 0.0001
Population × Adulthood temperature x Initial temperature	12	624.0	2.6	0.0031
Error	245	4945.8	-	-

Supplementary Table S5. Two-way analysis of variance (ANOVA) testing the effects of population, initial temperature and their interaction on the relative changes in the fat content (μg TAG equivalents/ fly) at three adulthood temperatures on the standard medium. *df* - degrees of freedom; SSQ - the sum of squares for each source of variation.

**Adulthood temperature**	**Source of variation**	***df***	**SSQ**	***F*-ratio**	***P*-value**
**18°C**	Population	3	1.3	33.0	< 0.0001
	Initial temperature	2	3.4	128.7	< 0.0001
	Population × Initial temperature	6	3.6	45.3	< 0.0001
	Error	80	1.1	-	-
**25°C**	Population	3	0.1	1.6	0.2060
	Initial temperature	2	0.7	24.9	< 0.0001
	Population × Initial temperature	6	3.5	38.9	< 0.0001
	Error	79	1.2	-	-
**29°C**	Population	3	0.2	11.2	< 0.0001
	Initial temperature	2	0.5	39.0	< 0.0001
	Population × Initial temperature	6	1.9	45.3	< 0.0001
	Error	80	0.6	-	-

Supplementary Table S6. Two-way analysis of variance (ANOVA) testing the effects of population, initial temperature and their interaction on the relative changes in the fat content (μg TAG equivalents/ fly) at three adulthood temperatures on the high protein medium. *df* - degrees of freedom; SSQ - the sum of squares for each source of variation.

**Adulthood temperature**	**Source of variation**	***df***	**SSQ**	***F*-ratio**	***P*-value**
**18°C**	Population	3	0.9	13.5	< 0.0001
	Initial temperature	2	0.7	15.6	< 0.0001
	Population × Initial temperature	6	0.9	6.6	< 0.0001
	Error	81	1.8	-	-
**25°C**	Population	3	0.1	3.0	0.0369
	Initial temperature	2	0.6	18.2	< 0.0001
	Population × Initial temperature	6	2.8	30.3	< 0.0001
	Error	82	1.2	-	-
**29°C**	Population	3	0.2	3.1	0.0317
	Initial temperature	2	0.3	9.1	0.0003
	Population × Initial temperature	6	0.4	4.1	0.0011
	Error	82	1.3	-	-

Supplementary Table S7. Two-way analysis of variance (ANOVA) testing the effects of population, developmental temperature and their interaction on the fat content (µg TAG equivalents/ fly) at eclosion. *df* - degrees of freedom; SSQ - the sum of squares for each source of variation.

**Source of variation**	***df***	**SSQ**	***F*-ratio**	***P*-value**
Population	3	81.0	1.2	0.3319
Developmental temperature	2	4968.8	106.7	< 0.0001
Population × Developmental temperature	6	1869.3	13.4	< 0.0001
Error	66	1537.1	-	-

Supplementary Table S8. *F*-test for parallelism for age-specific decrease in the fat content (μg TAG equivalents/ fly) standardized to the starting value (the fat content at eclosion). *ndf* – numerator degrees of freedom; *ddf* – denominator degrees of freedom.

**Population**	**Comparison**	***ndf***	***ddf***	***F*-ratio**	***P*-value**
**Oregon R**	18°C vs. 25°C	1	8	4.34	0.0707
	18°C vs. 29°C	1	8	7.45	0.0259
	25°C vs. 29°C	1	8	3.37	0.1036
w^{1118}	18°C vs. 25°C	1	8	0.27	0.6202
	18°C vs. 29°C	1	8	1.76	0.2212
	25°C vs. 29°C	1	8	1.16	0.3122
**Canton S**	18°C vs. 25°C	1	8	4.27	0.0726
	18°C vs. 29°C	1	8	7.31	0.0269
	25°C vs. 29°C	1	8	1.90	0.2053
**Denmark**	18°C vs. 25°C	1	8	0.00	0.9902
	18°C vs. 29°C	1	8	3.04	0.1193
	25°C vs. 29°C	1	8	3.51	0.0980

Supplementary Table S9. Two-way analysis of variance (ANOVA) testing the effects of population, temperature (24 h exposure) and their interaction on the fat content (µg TAG equivalents/ fly). *df* - degrees of freedom; SSQ - the sum of squares for each source of variation.

**Temperature**	**Source of variation**	***df***	**SSQ**	***F*-ratio**	***P*-value**
**29°C**	Population	3	1278.1	37.5	< 0.0001
	Temperature	1	50.2	4.4	0.0421
	Population × Temperature	3	4.4	0.1	0.9431
	Error	38	431.2	-	-
**31°C**	Population	3	1791.7	44.2	< 0.0001
	Temperature	1	33.3	2.5	0.1249
	Population × Temperature	3	50.7	1.3	0.3052
	Error	38	513.9	-	-
**33°C**	Population	3	1404.9	39.6	< 0.0001
	Temperature	1	236.8	20.0	< 0.0001
	Population × Temperature	3	32.0	0.9	0.4495
	Error	38	449.1	-	-

Supplementary Table S10. Two-way analysis of variance (ANOVA) testing the effects of population, thermal stress and their interaction on the fat content (µg TAG equivalents/ fly). *df* - degrees of freedom; SSQ - the sum of squares for each source of variation.

**Type of thermal stress**	**Source of variation**	***df***	**SSQ**	***F*-ratio**	***P*-value**
**Heat-shock (38°C, 45 min)**	Population	3	1867.0	41.7	< 0.0001
	Thermal stress	1	562.2	37.7	< 0.0001
	Population × Thermal stress	3	19.0	0.4	0.7376
	Error	32	477.7	-	-
**Cold exposure (0°C, 4h)**	Population	3	1075.6	32.2	< 0.0001
	Thermal stress	1	653.0	58.6	< 0.0001
	Population × Thermal stress	3	24.2	0.7	0.5441
	Error	36	401.1	-	-
**Cold exposure (4°C, 4h)**	Population	3	1404.6	45.5	< 0.0001
	Thermal stress	1	277.8	27.0	< 0.0001
	Population × Thermal stress	3	213.9	6.9	0.0009
	Error	34	350.2	-	-

Supplementary Table S11. Two-way analysis of variance (ANOVA) testing the effects of population, starvation (24 h) and their interaction on the fat content (µg TAG equivalents/ fly) at two time points (0.day – immediately after 24h starvation). *df* - degrees of freedom; SSQ - the sum of squares for each source of variation.

	**Source of variation**	***df***	**SSQ**	***F*-ratio**	***P*-value**
**0.day**	Population	3	332.1	15.8	< 0.0001
	Starvation	1	1403.5	199.7	< 0.0001
	Population × Starvation	3	26.5	1.3	0.3023
	Error	40	281.2	-	-
**5.day**	Population	3	537.3		< 0.0001
	Starvation	1	55.4		0.0063
	Population × Starvation	3	1.8		0.9652
	Error	40	266.9	-	-

Supplementary Table S12. Two-way analysis of variance (ANOVA) testing the effects of population, heat-shock (38ºC, 45 min) and their interaction on the fat content (µg TAG equivalents/ fly) at three time points (0.day – immediately after the heat-shock). *df* - degrees of freedom; SSQ - the sum of squares for each source of variation.

	**Source of variation**	***df***	**SSQ**	***F*-ratio**	***P*-value**
**0.day**	Population	3	1658.4	35.1	< 0.0001
	Heat-shock	1	25.0	1.6	0.2170
	Population × Heat-shock	3	14.4	0.3	0.8221
	Error	32	504.1	-	-
**5.day**	Population	3	1867.5	53.8	< 0.0001
	Heat-shock	1	1628.4	40.8	< 0.0001
	Population × Heat-shock	3	201.8	5.8	0.0023
	Error	38	439.5	-	-
**10.day**	Population	3	2803.3	86.4	< 0.0001
	Heat-shock	1	562.5	52.0	< 0.0001
	Population × Heat-shock	3	195.9	6.0	0.0019
	Error	37	400.1	-	-

Supplementary Table S13. Two-way analysis of variance (ANOVA) testing the effects of population, cold exposure (0ºC, 4 h) and their interaction on the fat content (µg TAG equivalents/ fly) at three time points (0.day – immediately after the cold exposure). *df* - degrees of freedom; SSQ - the sum of squares for each source of variation.

	**Source of variation**	***df***	**SSQ**	***F*-ratio**	***P*-value**
**0.day**	Population	3	1230.9	49.5	< 0.0001
	Cold exposure	1	4.3	0.5	0.4757
	Population × Cold exposure	3	50.5	2.0	0.1295
	Error	32	265.2	-	-
**5.day**	Population	3	2469.9	43.6	< 0.0001
	Cold exposure	1	525.9	27.8	< 0.0001
	Population × Cold exposure	3	248.8	4.4	0.0096
	Error	38	718.3	-	-
**10.day**	Population	3	2458.3	97.2	< 0.0001
	Cold exposure	1	240.5	28.5	< 0.0001
	Population × Cold exposure	3	302.7	12.0	< 0.0001
	Error	37	311.8	-	-

Supplementary Table S14. Two-way analysis of variance (ANOVA) testing the effects of population, exposure to different temperatures (‘Temperature’; 18°C, 25°C and 29°C) and their interaction on the fat content (µg TAG equivalents/ fly) at three time points (0.day – immediately after the exposure). *df* - degrees of freedom; SSQ - the sum of squares for each source of variation.

	**Source of variation**	***df***	**SSQ**	***F*-ratio**	***P*-value**
**0.day**	Population	3	1179.3	42.9	< 0.0001
	Temperature	2	2314.0	126.1	< 0.0001
	Population × Temperature	6	98.9	1.8	0.1149
	Error	60	550.4	-	-
**5.day**	Population	3	754.2	31.4	< 0.0001
	Temperature	2	448.1	28.0	< 0.0001
	Population × Temperature	6	51.6	1.1	0.3878
	Error	60	479.8	-	-
**10.day**	Population	3	667.1	38.8	< 0.0001
	Temperature	2	302.6	26.4	< 0.0001
	Population × Temperature	6	44.0	1.3	0.2792
	Error	66	378.5	-	-

Supplementary Table S15. Starvation survival analyses (see Fig.5). *df* - degrees of freedom

**Treatment**	**Population**	**Log-rank test**			**Wilcoxon test**		
		**Chi-square**	***df***	***P*-value**	**Chi-square**	***df***	***P*-value**
**Heat-shock (38°C, 45 min)**	Oregon R	194.99	1	< 0.0001	180.32	1	< 0.0001
	w^{1118}	175.56	1	< 0.0001	169.58	1	< 0.0001
	Canton S	77.46	1	< 0.0001	84.62	1	< 0.0001
	Denmark	56.40	1	< 0.0001	64.43	1	< 0.0001
**Cold-shock (0°C, 4 h)**	Oregon R	91.10	1	< 0.0001	79.81	1	< 0.0001
	w^{1118}	125.04	1	< 0.0001	129.72	1	< 0.0001
	Canton S	53.41	1	< 0.0001	61.99	1	< 0.0001
	Denmark	38.51	1	< 0.0001	30.94	1	< 0.0001
**18°C vs 29°C (8 days)**	Oregon R	34.36	1	< 0.0001	31.54	1	< 0.0001
	w^{1118}	55.00	1	< 0.0001	53.86	1	< 0.0001
	Canton S	44.80	1	< 0.0001	38.27	1	< 0.0001
	Denmark	17.29	1	< 0.0001	16.50	1	< 0.0001

Supplementary Table S16. Summary of transgenic *Drosophila* strains used in this study; BDSC refers to Bloomington Drosophila Stock Center.

Short name	Genotype	Reference/Source	Internal stock number
da-GS	w^{1118}*; da-GS*	Tricoire *et al.* [4]	MGF 1663
FBI-26-GS	*P{Switch1}FBI-26; UAS-GFP*	Roman *et al.*[5]	RKF 1045
ts-FB-Gal4	*y*w* ; P{w[+mW.hs]=GawB}FB P{w[+m*] UAS-GFP 1010T2}#2; P{w[+mC]=tubPGAL80[ts]}2*	Beller *et al.*[6]	RKF 805
Lpp-Gal4	*w*; +/+; P{Lpp-GAL4.B}/TM3, Sb**	Brankatschk and Eaton[2]	RKF 1421
UAS-StingerII	*+; UAS-StingerII*	Barolo *et al.* [3]	RKF 1171
UAS-*hid*	*+; P{UAS-hid[14]} / CyO*	Received from M. Hoch	RKF 174
UAS-*Diap1*	*w[*]; P{w[+mC]=UAS-DIAP1.H}3*	BDSC 6657	PKF 1725
Hsf RNAi (1)	*y[1] v[1]; P{y[+t7.7] v[+t1.8]=TRiP.JF02415}attP2/TM3, Sb[1]*	BDSC 27070	PKF 1726
Hsf RNAi (2)	*y[1] v[1]; P{y[+t7.7] v[+t1.8]=TRiP.GL00698}attP2*	BDSC 41581	PKF 1727
Hsp83 RNAi (1)	*y[1] sc[*] v[1]; P{y[+t7.7] v[+t1.8]=TRiP.HMS00796}attP2*	BDSC 32996	PKF 1728
Hsp83 RNAi (2)	*y[1] sc[*] v[1]; P{y[+t7.7] v[+t1.8]=TRiP.HMS00899}attP2*	BDSC 33947	PKF 1729
Hsp70Aa; Hsp70Ab RNAi	*y[1] v[1]; P{y[+t7.7] v[+t1.8]=TRiP.HMS02475}attP40*	BDSC 42639	PKF 1730
Hsp70Ba RNAi (1)	*y[1] sc[*] v[1]; P{y[+t7.7] v[+t1.8]=TRiP.GLV21037}attP2*	BDSC 35672	PKF 1731
Hsp70Ba RNAi (2)	*y[1] sc[*] v[1]; P{y[+t7.7] v[+t1.8]=TRiP.HMS02661}attP40*	BDSC 43289	PKF 1732
Hsp23 RNAi	*y[1] sc[*] v[1]; P{y[+t7.7] v[+t1.8]=TRiP.HMS02745}attP40*	BDSC 44029	PKF 1733

Supplementary References

1. Gáliková, M. *et al.* Energy homeostasis control in *Drosophila* Adipokinetic hormone mutants. *Genetics* **201,** 665–683, doi:10.1534/genetics.115.178897, (2015).
2. Brankatschk, M. & Eaton, S. Lipoprotein particles cross the blood-brain barrier in *Drosophila*. *J. Neurosci.* **30,** 10441–10447, doi:10.1523/JNEUROSCI.5943-09.2010 (2010).
3. Barolo, S., Carver, L. A. & Posakony, J. W. GFP and beta-galactosidase transformation vectors for promoter/enhancer analysis in *Drosophila*. *BioTechniques* **29,** 726–732 (2000).
4. Tricoire, H. *et al.* The steroid hormone receptor EcR finely modulates *Drosophila* lifespan during adulthood in a sex-specific manner. *Mech. Ageing Dev.* **130,** 547–552, doi:10.1016/j.mad.2009.05.004 (2009).
5. Roman, G., Endo, K., Zong, L. & Davis, R. L. P[Switch], a system for spatial and temporal control of gene expression in *Drosophila melanogaster*. *Proc. Natl. Acad. Sci. U.S.A.* **98,** 12602–12607, doi:10.1073/pnas.221303998 (2001).
6. Beller, M. *et al.* PERILIPIN-dependent control of lipid droplet structure and fat storage in *Drosophila*. *Cell Metab.* **12,** 521–532, doi:10.1016/j.cmet.2010.10.001 (2010).
